# An Overview of Systemic Lupus Erythematosus (SLE) Pathogenesis, Classification, and Management

**DOI:** 10.7759/cureus.30330

**Published:** 2022-10-15

**Authors:** Muhammad Atif Ameer, Haroon Chaudhry, Javaria Mushtaq, Osama S Khan, Maham Babar, Tehmina Hashim, Saima Zeb, Muhammad Ali Tariq, Sridhar Reddy Patlolla, Junaid Ali, Syeda Nafeesa Hashim, Sana Hashim

**Affiliations:** 1 Department of Medicine, Punjab Rangers Teaching Hospital, Lahore, PAK; 2 Internal Medicine, Suburban Community Hospital, East Norriton, USA; 3 Cell Biology and Neurosciences, Rowan University, Stratford, USA; 4 Surgery, Khyber Medical University, Peshawar, PAK; 5 Department of Internal Medicine, Khyber Medical University, Peshawar, PAK; 6 Pathology, Batterjee Medical College, Jeddah, SAU; 7 Biochemistry, Northwest School of Medicine, Peshawar, PAK; 8 Angiocore Lab, Medstar Washington Hospital Center, Washington D.C., USA; 9 Department of Medicine, Khyber Teaching Hospital, Peshawar, PAK; 10 Medicine, Batterjee Medical College, Jeddah, SAU; 11 Medicine and Surgery, Batterjee Medical College, Jeddah, SAU

**Keywords:** constitutional symptoms and sle, comorbids of sle, preclinical lupus, lupus nephritis, pathogenesis of sle, sle, european alliance of associations for rheumatology (eular) classification, sle and lupus nephritis, systemic lupus erythematosis

## Abstract

Systemic lupus erythematosus (SLE) is a complex autoimmune disease with multisystem involvement. It is multifactorial and involves epigenetic, genetic, ecological, and environmental factors. Primarily it leads to activation of both innate and adaptive immunity, which consequently leads to autoreactive B cell activation by T cells and leads to immune complexes deposition in tissues leading to an autoimmune cascade that may be limited to the single organ or can cause a widespread systemic involvement. SLE is heterogeneous in presentation, with a broad spectrum of clinical manifestations ranging from clinically mild self-resolving symptoms to severe life-threatening organ involvement. Clinical and serological heterogeneity are critical features in SLE, posing a significant challenge in its diagnosis. Antinuclear antibodies (ANA) are the telltale serological marker in more than 95% of SLE patients. The improved set of European Alliance of Associations for Rheumatology (EULAR) classification enabled accurate diagnosis of SLE. The treatment focuses on remission, preventing organ damage, and improving the overall quality of life.

## Introduction and background

Systemic lupus erythematosus (SLE) is a multisystem chronic autoimmune disease with a relapsing and remitting course. Its prevalence is higher in women of childbearing age, with a female predominance of 9:1 [1]. The exact etiology of this disease is not understood well. However, it has been demonstrated that environmental and genetic factors interact to trigger immune responses resulting in the excessive production of pathogenic autoantibodies by the B cells and cytokines dysregulation leading to tissue and organ damage. SLE is characterized by the presence of antibodies to nuclear and cytoplasmic antigens [2].

Furthermore, other autoantibodies might be present in SLE patients, such as anti-Scl-70 antibodies (present in systemic sclerosis), anti-La, and anti-Ro antibodies (present in Sjogren disease), anticardiolipin antibodies, and anti-phospholipid antibodies thus indicating a wide association between SLE and other autoimmune diseases [3]. This condition has a broad spectrum of clinical features ranging from mild cutaneous involvement to severe organ damage, such as kidney failure, pulmonary hypertension, and cardiac failure. The diagnosis of SLE is based on clinical and laboratory findings. The improved classification criteria used by the European League Against Rheumatism (EULAR) and the American College of Rheumatology (ACR) [4] serve as the most advanced and precise criteria to date. The management of SLE is challenging and requires a multidisciplinary approach. The treatment algorithm is based on the severity of the diseases and the organs involved. Though the disease affects multiple systems, the course may vary in individuals depending on the severity, the number of flare-ups, and remission. Life expectancy may be reduced depending on the significant organ involvement, such as kidneys, lungs, and heart. Otherwise, with close follow-up, around 80% to 90% of patients with SLE may have an average life expectancy [5].

A comprehensive search of databases and search engines, including PubMed/Medline and Google Scholar, was performed using appropriate keywords, including systemic lupus erythematosus, the pathogenesis of SLE, the role of B/T cells in SLE, and classification criteria of SLE. In addition, articles that could contribute to the pathogenesis, classification, and diagnosis management were included in the review. The information gathered from the literature search is integrated with clinical expertise and presented as a comprehensive review.

## Review

Epidemiology

Globally, the reported incidence and prevalence of SLE differ significantly by geography, with North America reporting the highest incidence and prevalence, Africa reporting the lowest incidence, and Australia reporting the lowest prevalence. Age, gender, and ethnicity play a significant role in determining the clinical outcome and management of the disease. SLE is more prevalent in the female population, but its course is more critical and expeditious in men, which culminates in a bad prognosis [6]. This disparity can be attributed to the environmental surroundings and genomic differences. 

The current incidence rate is 6.73 cases per 100,000 per annum in the Caucasian population and 31.4 cases per 100,000 per annum in the African-American population. The prevalence rate among the U.S. black population is 517 per 100,000, while it is 134 per 100,000 among U.S. Caucasians and Europeans [6,7].

SLE is seen mainly in women during the childbearing age between 15-44 years [8], with a female predominance of 9:1, making SLE one of the most gender-differentiated autoimmune diseases [7]. SLE, a common diagnosis during reproductive age, suggests hormonal influence in its pathogenesis, which also presents a host of medical and psychosocial challenges that affect family planning and pregnancy. The racial tendencies in SLE indicate that this disease primarily affects non-Caucasian individuals. In the United States, SLE is more frequent among African American, Hispanic, and Asian populations as compared to the Caucasian population. Its occurrence is three to four times higher among African-American women [8].

Pathogenesis

The pathogenesis of SLE includes a complex interaction between the exposome (environmental influence) and genome to produce an epigenetic change that alters the expression of specific genes that contribute to disease development. Exposure to environmental factors such as UVB radiation, infections, and toxins triggers a loss of immune tolerance in genetically susceptible individuals and leads to aberrant activation of autoimmunity [2]. Exposure of self-antigens to the immune cells, possibly from an increased apoptotic cell load, initiates a feed-forward loop between innate and adaptive immunity. The ensuing production of autoantibodies and immune complexes, autoreactive T cells and B cells, complement activation, and cytokine release result in widespread tissue damage, manifesting as the clinical picture of SLE [1]. This review focuses on SLE's genetic susceptibility, environmental influences, and immunopathogenesis.

Genetic Susceptibility

In the last decade, a genome-wide association study (GWAS) has mapped >90 SLE susceptibility loci, with many single nucleotide polymorphisms acting additively. In addition, rare monogenic forms of SLE have also been reported [9]. Among the 730 SLE-associated polymorphisms, 21 lead to amino acid change, 484 exist within gene coding regions, and the rest are intergenic, suggesting a significant effect on gene regulation instead of protein sequence [10]. Most SLE risk loci are located within or near genes that encode products functioning in the clearance of immune complexes (IC), lymphocyte signaling, and type I interferon (IFN-I) signaling. The SLE risk genes reviewed here are grouped in the context of significant disease pathways; however, due to their diverse functions, these genes could play a role in disease pathogenesis through multiple mechanisms [11-12].

T/B Cell Signaling

The susceptibility genes involved in aberrant T/B cell signaling in SLE encode adaptor molecules, kinases, and cytokines that regulate T/B-cell activation, proliferation, and interaction. For example, the class II human leukocyte antigen (HLA) region encodes molecules involved in antigen presentation [2,13]. The upregulated surface expression of these molecules leads to a hyperactive immune response. HLA-DR2 and HLA-DR3 alleles are associated with SLE susceptibility and autoantibody production. Protein tyrosine phosphatase non-receptor type 22 (PTPN22) is another gene that encodes a tyrosine phosphatase that alters T-cell receptor (TCR) and B-cell receptor (BCR) signaling leading to enhanced B-cell autoreactivity in SLE. Similarly, C-terminal Src kinase (CSK) is another protein-encoding gene that encodes C-Src tyrosine kinase and BANK1, which encodes an adaptor/scaffold protein associated with altered B-cell activation. Transcription factors encoded by the SLE susceptibility genes that regulate lymphocyte differentiation and proliferation include ETS1, IKZF1, IKZF2, IKZF3, RAG1, RAG2, FASLG, FAS, SHOC2, and KRAS [14].

Role of T Cells

T cells play a significant role in SLE pathogenesis, driving inflammation by secretion of pro-inflammatory cytokines, inducing B cells to generate autoantibodies, and maintaining disease via a pool of autoreactive memory T cells. However, the ratios of some T cell subsets and their function are abnormal in patients with SLE [15].

T follicular helper (Tfh) cells are essential for germinal center induction, proliferation, isotype-switching, and somatic hypermutation. In addition, these cells produce cytokine IL-21, which induces B cell differentiation into memory B cells and antibody-generating plasmablasts. Pathological expansion of Tfh cells in SLE is directed by the interaction of the Tfh cells with the OX40 ligand expressed on myeloid antigen-presenting cells [16]. The expression of the OX40 ligand is induced by TLR7 activation from circulating RNA-containing immune complexes. This pathologic expansion of the TFH cell subset contributes to enhanced antibody production and loss of tolerance in SLE patients [15-17].

Regulatory T (Treg) cells are a unique T cell subset population that suppresses the immune response and maintains self-tolerance, suppressing autoreactive lymphocytes in healthy individuals. The development of Treg cells is dependent on IL­2 activity. In SLE, an imbalanced T cell cytokine profile characterized by decreased IL­2 leads to impaired Treg cell development and function [18]. Reduced expression of IL­2 in T cells is caused by low levels of the transcription factor activator protein 1 (AP-1), which ultimately fuels SLE's development. IL2 also plays a role in restricting the expression of IL­17, which is pro-inflammatory and its elevated levels in SLE contribute to local tissue damage [19].

Role of B Cells

B cells contribute to the pathogenesis of SLE through their response to antigens and autoantibody production. The pathways implicated in the aberrant activation of B cells include the toll-like receptor (TLR) pathway, stimulation via beta cell-activating factor (BAAF), and B-cell receptor (BCR) mediated activation. The stimulation of B cells through the TLR pathway promotes loss of tolerance [2].

Transitional B cells are susceptible to TLR9 stimulation and produce autoreactive marginal zone B cells, as seen in SLE patients. An impairment of B cell tolerance can also occur through B cell stimulation from cytokines, particularly BAFF. SLE patients with high levels of BAFF exhibit significantly higher levels of anti-dsDNA, anti-histone, and anticardiolipin antibodies [17].

Furthermore, BCR is a crucial regulator of negative and positive selection, and continuous BCR sensing is necessary for B cell survival in healthy individuals. SLE patients with polymorphisms of the c-Src tyrosine kinase (Csk) gene exhibit a higher level of BCR-mediated activation of B cells and a higher concentration of serum IgM levels [9,20].

Pro-survival BAFF signaling counterbalances the pro-apoptotic signals induced by BCR. However, an imbalance of these signals in SLE leads to a loss of tolerance, and autoantibody production eventually contributes to the disease pathogenesis [21]. 

Aberrant Apoptotic Cell Clearance

Dysregulation of apoptosis and nuclear debris clearance contributes to an increase in autoantigen exposure. Several pathways evolve to prevent immune activation in response to endogenous cellular debris, but these mechanisms are impaired in SLE [22]. Hence, increased survival of defective lymphocytes is thought to be one of the mechanisms contributing to pathogenesis. Furthermore, inadequate clearance due to faulty apoptosis of IgG2- and IgG3-containing complexes also results in defective clearance. Usually, Tyro-3, Axl, and Mer (TAM) receptors are expressed by phagocytes, macrophages, and natural killer cells in rheumatological autoimmune diseases [23]. The downstream activation of TAM receptors promotes the phagocytosis of apoptotic cells. In addition, it inhibits the signal transducer and activator of transcription 1 (STAT1) and the nuclear factor kappa-light-chain-enhancer of the activated B cell (NF-κB) pathway. In animal studies, TAM knockout has been associated with the presence of anti-dsDNA antibodies, anti-phospholipid antibodies, and SLE-like manifestations, such as arthritis, vasculitis, and the deposition of immunoglobulin G in renal glomeruli [24].

C-reactive protein (CRP) can bind to the nuclear antigens of apoptotic bodies and neutralize their immunogenicity potential. Single nucleotide polymorphism of the CRP gene, mainly CRP4, is linked with low serum CRP levels and SLE. In addition, DNase I contributes to the degradation of chromatin released from apoptotic cells, reducing exposure of nuclear antigens to the immune cells. A loss-of-function variant in the DNASE1L3 gene is associated with a familial form of SLE [14]. However, the exact cause of defective clearance of apoptotic bodies is still far from understood.

Role of the Complement System

Complement dysfunction is proposed to accelerate several steps in the pathogenic pathways of SLE, such as impaired clearance of apoptotic debris and IC, increased autoreactive CD+8 T cell activity, and tissue damage by activation of the inflammatory cascade in organs with IC deposition [25]. C1q typically assists in the removal of apoptotic material and immune complexes and inhibits the CD8+ T cell response to self-antigens by modulating their mitochondrial metabolism. Patients with C1q homozygous deficiency develop autoantibodies and a lupus-like syndrome, evidently due to the inability to eliminate apoptotic cells [26].

Clearance of Apoptotic Cells and Immune Complexes

Exposure to self-antigens due to impaired clearance of apoptotic cells triggers the initiation of an autoimmune response. In addition, the impaired clearance of IC formed from autoantibodies bound to antigens can amplify the inflammatory response [27]. HLA class III region genes encode products relevant to this pathway; C4A, C4B, C1QA, C1QB, and C1QC mutations are associated with monogenic forms of SLE [28].

Role of Toll-Like Receptors

TLRs abnormality in SLE has been widely documented. B cell lymphocytes associated with TLRs' mechanistic dysfunction play a significant role in the pathogenesis of the disease [29]. TLRs are nucleic acid recognition receptors that trigger an inflammatory response upon activation by nuclear antigens contained in IC or apoptotic debris. TLRs are germline-encoded receptors that recognize certain microorganisms and are usually the first line of defense against them, and primarily respond to endocytosed nucleic acids. Downstream activation of TLRs leads to activation of two transcription factors, interferon regulatory factor 3 (IRF3) and nuclear factor‑κB (NF-κB), which induces the expression of type I interferon (IFN), which plays a central role in disease pathogenesis [9,17]. Overexpression of TLR7 (receptor for single-stranded RNA) is associated with the production of RNA-reactive autoantibodies and type IFN I production. Overexpression of TLR9 (receptor for DNA containing unmethylated CpG sequence motifs) is linked with elevated levels of anti-dsDNA antibodies and high disease burden [17].

Type I Interferon Signaling

Over half of the identified SLE-susceptibility genes encode proteins linked to IFN-I production or response. Toll-like receptor 7 (TLR7) overexpression is a well-known driver of increased IFN-I production and pathogenesis of SLE [30]. In the murine or mouse experimental model, the absence of type 1 interferon receptor protects against the development of SLE. Other genes mapped to this pathway include miR146a, RNASEH2C, SLC15A4, and IRAK1 [31].

Environmental factors and their influence

The association of SLE incidence with exposure to silica, cigarette smoke, oral contraceptives, ultraviolet B (UVB), certain drugs, and Epstein-Barr virus (EBV) infection has been well established by epidemiological studies. Potential biologic mechanisms for these associations include increased oxidative stress, inflammatory cytokine upregulation, systemic inflammation, and epigenetic modifications [12]. Specific environmental exposure and its proposed influence on the pathogenesis of SLE are reviewed here.

Particulate Exposure

Experimental studies suggest that crystalline silica induces cellular apoptosis and the release of intracellular antigens. Other disease mechanisms include increased activity of pro-inflammatory cytokines, oxidative stress, and diminished regulatory T-cell activity. In murine models, silica exposure is linked with higher serum autoantibodies and immune complexes [32]. Exposure to toxic components from cigarette smoke (e.g., nicotine, polycyclic aromatic hydrocarbons, carbon monoxide, and free radicals) can induce oxidative stress and directly damage endogenous proteins and DNA, leading to genetic mutations that potentially induce autoimmunity and augment the production of pro-inflammatory cytokines [33]. A meta-analysis of studies on smoking and SLE risk revealed that smokers had an increased risk of developing SLE (OR 1.5; 95% CI 1.09, 2.08) compared to non-smokers [34].

Ultraviolet B Exposure

UVB exposure induces reactive oxygen species and upregulates pro-inflammatory cytokines expression, such as IFN-α, IL-1, IL-6, and TNF-α. IFNs are involved in developing UVB-induced inflammatory skin lesions in patients with SLE. UVB also upregulates intracellular adhesion molecules (e.g., ICAM-1 and LAF-1) and increases the secretion of IL-8 and chemokine ligands (e.g., CCL5, CCL20, and CCL22), which recruit immune cells to areas of inflammation [12]. Another disease development mechanism is UVB-induced DNA hypomethylation in CD4+ T cells, promoting autoreactivity and autoantibody production. Furthermore, UVB exposure leads to impaired clearance of apoptotic cells [35,36].

Epstein-Barr Virus Infection

EBV seropositivity rates are significantly higher among SLE patients than in their age-matched controls [36]. Potential mechanisms involve genetic insufficiencies that result in poor infection control and more frequent reactivation of latent EBV infection. High viral loads, elevated levels of EBV IgA antibodies, and defective EBV-specific T cells in SLE patients indicate a lack of adequate infection control [37,38]. The primary genetic predisposing factors are deficiencies in the complement pathway, components of IFN pathways, and specific major histocompatibility complex (MHC) alleles. Additionally, due to widespread latent infection and reactivation, an increased number of EBV-infected cells upon apoptosis will initiate an innate and adaptive immune response against released cellular antigens and EBV antigens. Poor control of EBV infection may thus be a contributing factor to the development of SLE. Another mechanism by which EBV can contribute to the development of SLE is molecular mimicry. Antibodies against EBV nuclear antigen (EBNA-1) cross-react with SLE-associated autoantigens. In susceptible individuals, the immune response against EBNA-1 leads to the generation of cross-reactive antibodies, followed by epitope spreading, which eventually results in the development of SLE. Other SLE-related viral associations include cytomegalovirus, parvovirus B19, and hepatitis B virus vaccine [39].

Drug Exposure

Drugs implicated in developing SLE are hydralazine, procainamide, isoniazid, minocycline, and TNF-α inhibitors. Procainamide and hydralazine are associated with the highest risk of inducing SLE, with 30% and 5% to 10%, respectively. Some proposed mechanisms of drug-induced lupus are genetic predisposition, drug biotransformation, and epigenetic changes in immune cells. Procainamide, hydralazine, and isoniazid are predominantly metabolized by acetylation utilizing N-acetyltransferase enzymes. Slow acetylators with genetic deficiency of N-acetyltransferase are prone to autoantibodies accumulation after exposure to these drugs [40]. In addition, some studies have suggested an association of HLA-DR2, HLA-DR3, C4A, and C4B null complement alleles with drug-induced lupus. These drugs may also inhibit the complement component C3, hindering the clearance of immune complexes. Biotransformation, another potential mechanism of drug-induced lupus, focuses on the oxidative metabolism of drugs within neutrophils. It is hypothesized that the oxidative metabolism of procainamide and isoniazid releases several toxic metabolites along with myeloperoxidase and reactive oxygen species, eliciting a cytotoxic effect [41]. Biotransformed drugs and their metabolites have been reported to alter the epigenetic properties of immune cells. Hydralazine and procainamide inhibit T-cell DNA methylation, causing increased lymphocyte function-associated antigen 1 (LFA-1) expression, which consequently induces autoreactivity. The innate immune response has also been implicated in developing drug-induced lupus. Neutrophil extracellular traps (NETs) are secreted by activated neutrophils and contain nuclear DNA and cytosolic proteins. Procainamide and hydralazine promote NET formation via triggering neutrophil muscarinic receptors, inducing an autoimmune response from auto-antigen exposure [40].

Hormonal Influences

Sex hormones are known to affect the functioning of the immune system, and they play a role as triggers or protectors in the development of SLE. Studies have shown an increased risk of SLE associated with exposure to estrogen, whereas progesterone and testosterone play a protective role by counteracting the effects of estrogen. Additionally, the influence of sex hormones on disease activity is evident in exacerbations during puberty, pregnancy, and post-partum periods. Estrogen activity may contribute to disease development by increasing the production of type 1 interferon (IFN), augmenting survival of auto-reactive B cells with the production of anti-dsDNA antibodies, dysregulation of T-reg cells, modulation of TLR pathways, and dendritic cell development [42].

Clinical presentation

SLE exhibits a broad spectrum of presentations ranging from mild symptoms to severe, life-threatening conditions. Adults diagnosed before 50 years of age usually present with cutaneous symptoms (malar rash) and renal abnormalities (lupus nephritis), displayed higher 10-year survival, and reported using more immunosuppressive therapy than patients getting diagnosed after 50 years of age [43]. Various factors such as age, race, gender, genetic premise, and socioeconomic status also influence the timeframe of presentation and therapy initiation [8]. Hence the clinical presentation can vary drastically, and a high level of suspicion is needed for early diagnosis and treatment of these patients.

Preclinical lupus (PL) is a phase in developing SLE when the patient is at higher risk of developing SLE but is found asymptomatic on presentation. However, autoantibodies are mostly detectable in these patients' serum [44]. Antinuclear antibody (ANA), hematological and immunological disorders, arthritis, and cutaneous manifestations were among the most presented symptoms of PL syndrome. Therefore, a significant proportion of preclinical lupus (approximately 10% to 20%) often transitions to SLE. Most PL patients are treated with steroids and other immunosuppressive therapies such as azathioprine and methotrexate. The most prevalent clinical presentations are summarized below (Table 1).

**Table 1:** Most commonly encountered signs and symptoms in the patients of systemic lupus erythematosus HTN: hypertension

System Involvement	Clinical presentation
Musculoskeletal	Jaccoud's arthropathy, arthralgia, arthritis, synovitis, tenosynovitis, myositis
Central and peripheral nervous system	Central Nervous system: Neuropsychiatric lupus, lupus cerebritis (seizure, headache), aseptic meningitis. Peripheral Nervous system: Transverse myelitis, mononeuritis multiplex, peripheral neuropathy, small fiber neuropathy, autonomic neuropathy. Additionally, delirium, and psychosis are also present
Gastrointestinal	Ascites, peritonitis, oral ulcers, esophageal dysmotility, protein-losing enteropathy
Hematological	Anemia, microangiopathic hemolytic anemia, thrombocytopenia
Pulmonary	Pleuritis, pulmonary arterial HTN, interstitial lung disease, pleural effusion
Cardiovascular	Libman-sacks-endocarditis, pericarditis, myocarditis
Renal	Proteinuria, hematuria, and glomerulonephritis

Cardiovascular System

All three layers of the heart, namely the pericardium, myocardium, and endocardium, and often, coronary circulation, may be affected in SLE. The frequently seen manifestations include cardiomyopathy, valvular diseases, rhythm discrepancies, and heart failure. The most prevalent cardiac manifestation is pericarditis secondary to exudative pericardial effusions [45].

Cutaneous Lupus

Around 90% of patients develop skin manifestations during the SLE course. It has different types with distinct characteristics. It includes acute cutaneous lupus erythematosus (ACLE), subcutaneous lupus erythematosus (SCLE), and chronic cutaneous lupus erythematosus (CCLE) (Table 2) [46]. Raynaud's phenomena, alopecia, and vasculitis fall under non-lupus-specific symptoms. One of the most well-established manifestations of the disease is mucocutaneous involvement. Painless oral ulcers start growing as erythema, macule, petechiae, and erosions and eventually form ulcers. Photosensitivity is a very distinguishing feature of SLE. UV light exposure and inflammatory reactions are considered the main etiologic reason for photosensitivity [35,47]. Butterfly rash, also known as malar rash, is a pathognomonic feature of acute cutaneous lupus erythematosus. It involves sun-exposed areas such as the cheeks and nasal bridge. The discoid rash (round or coin-shaped) is another common skin finding in SLE patients. Livedo reticularis is a reticular, lace-like, or net-like pattern, often purplish, seen on the extremities of SLE patients.

**Table 2:** Common dermatologic manifestations of systemic lupus erythematosus

Types	Clinical presentation
Acute Cutaneous Lupus Erythematosus (ACLE)	Hallmark: Malar or butterfly rash, erythematous raised pruritic rash, nasolabial folds are spared.
Subcutaneous Lupus Erythematosus (SCLE)	Photosensitive, widespread, non-indurated rash
Chronic cutaneous lupus erythematosus (CCLE)	Discoid lupus erythematosus (DLE) is the most common type

Gastrointestinal System

A vast array of gastrointestinal (GI) symptoms are also observed in SLE, including flatulence, diarrhea, abdominal cramps, hematemesis, gastric atony, duodenal and jejunal ileus, chronic ulcerative colitis, oral ulcers, esophageal dysmotility issues, protein-losing enteropathy, and lupus enteritis. Moreover, mesenteric vessel thrombosis, Budd-Chiari syndrome, and hepatic veno-occlusive disease also occur secondary to SLE and anti-phospholipid antibody syndrome [48].

Hematological System

Around 18% to 80% of patients with SLE suffer from anemia. Anemia of chronic disease is the most prevalent type encountered in SLE. Microangiopathic hemolytic anemia, iron deficiency anemia, coomb's positive autoimmune hemolytic anemia, red blood cell aplasia, anemia secondary to chronic renal disease, and pancytopenia are also seen in SLE. Autoimmune cytopenia is not infrequent in SLE owing to the presence of antigens in the blood vessel compartment, resulting in more production of antibodies [49,50].

Musculoskeletal System

Musculoskeletal manifestations such as arthralgia and arthritis are present in 80% to 90% of patients with SLE. Though any joint can be affected most commonly, there is symmetrical involvement of small joints such as hands, wrists, and knees. The condition is aptly named lupus arthritis [51]. It is also known as Jaccoud's arthropathy. SLE arthritis might have a similar presentation to rheumatoid arthritis, including ulnar deviation and subluxation of the metacarpophalangeal joints, and the term "ruphus" has been coined to represent this condition. Rarely, cases of avascular necrosis of the hip joint with bilateral involvement have also been reported [52].

Nervous System

The central and peripheral nervous system involvement and psychiatric symptoms are often seen in SLE. Often headache is the most frequently encountered symptom. Additionally, there is an increased risk for ischemic stroke in SLE patients compared to the general population. Cognitive dysfunction is another significant concern in SLE patients, as different studies showed a cognitive decline in these patients. Seizures, aseptic meningitis, demyelinating syndrome, and movement disorder are other CNS manifestations. Complications associated with the parasympathetic nervous system include autonomic neuropathies, mononeuritis multiplex, and central and peripheral neuropathies. Psychiatric symptoms include anxiety, depression, and psychosis [53].

Pulmonary System

One of the most frequently seen pulmonary symptoms includes pleuritis, pleural effusion, acute reversible hypoxemia, pulmonary embolism, obstructive lung disease, and upper airway disease. Pulmonary arterial hypertension is another grave complication of SLE. Other pulmonary conditions associated with SLE are lupus pneumonitis, interstitial lung disease, usual interstitial pneumonia and diffuse alveolar hemorrhage, and pulmonary embolism [54].

Renal System

One of SLE's most prevalent and recognized clinical presentations is lupus nephritis. It is one of the earliest manifestations of SLE and occurs in around 50% of patients [55]. Interstitial nephritis and thrombotic angiopathy are among the other renal manifestations which can be attributed to a surge of inflammatory cytokine profiles, for example, interleukins (IL-1, IL-6, IL-17, IL-18), tumor necrotic factor, Th1 and Th2 cytokines [56].

Initially, proteinuria usually raises the suspicion of renal involvement. A wide range of manifestations (including mild presentation of sub-nephrotic proteinuria) may lead to diffuse involvement of renal structures, resulting in progressive glomerulonephritis and end-stage renal disease. Important signs and symptoms of renal involvement are lupus nephritis, including hematuria, raised creatinine, lower limb edema, anasarca, and new onset of hypertension.

Lupus nephritis (LN) is classified into six categories based on renal biopsy results. It includes glomerular immune complexes deposition, infiltration of renal parenchyma by T cells and macrophages, and activation of toll-like receptors leading to elevated levels of antibodies and interferons [57]. Different stages of renal manifestations of SLE and their prognosis are listed below (Table 3).

**Table 3:** Classes of lupus nephritis and their prognosis

Stages of Lupus Nephritis	Prognosis
Class I: Minimal mesangial lupus nephritis	Excellent prognosis
Class II: Mesangial proliferative lupus nephritis	Excellent prognosis
Class III: Focal lupus nephritis	Poor outcome
Class IV: Diffuse segmental nephritis	Poor outcome
Class V: Membranous lupus nephritis	Prognosis is favorable but with certain complications: Thromboembolism
Class VI: Advanced sclerosing lupus nephritis	Poor outcome as most symptoms are of irreversible injury [46]

Diagnosis

SLE presents with a wide array of clinical manifestations and an expansive profile of autoantibodies. This clinical and serological heterogeneity makes it a great challenge to reach an accurate diagnosis. Therefore, physician acumen plays a pivotal role in diagnosing SLE since various clinical features, serological findings, imaging, and histopathology must be considered simultaneously. Here we have discussed the widely used classification criteria of SLE and the biomarkers crucial in the diagnostic approach for SLE.

Classification Criteria

Several classification criteria for SLE have been formulated with the primary goal of grouping individuals for clinical studies. Furthermore, these can provide a backbone for the diagnostic approach in an individual patient [4]. The three most accepted classification criteria exist for SLE as follows: 1. the 1997 ACR (American College of Rheumatology), 2. the 2012 SLICC (Systemic Lupus International Collaborating Centers), and 3. the 2019 EULAR/ACR (European League Against Rheumatism/American College of Rheumatology).

Each criterion is built on the previous sets by refining, adding, or new information. The major limitation of the 1997 ACR as diagnostic criteria was a low sensitivity of 83%. According to this classification, one in six patients of SLE would not be correctly classified, with sensitivity dropping to 66% early in the disease, because criteria items may need time to accumulate during disease, which was a further limitation to using 1997 ACR as a diagnostic criterion. To rectify this, the 2012 SLICC was introduced with improved sensitivity of 97% and an increase in sensitivity to 84% early in the disease. However, the specificity decreased to 84%, whereas ACR criteria specificity was 93% [58].

The 2019 EULAR/ACR classification criteria were devised to maintain the high specificity of the ACR criteria and the high sensitivity of the SLICC criteria. Validation cohorts suggested this goal was reached with a specificity of 93% and a sensitivity of 96%. The 2019 EULAR/ACR classification criteria for SLE include a positive ANA test followed by additive weighted criteria that are grouped into seven clinical (constitutional, hematological, musculoskeletal, mucocutaneous, serosal, renal, neuropsychiatric) and three immunological (SLE-specific antibodies, anti-phospholipid antibodies, complement proteins,) domains; weighted from 2 to 10. Patients accumulating ≥10 points are classified as having SLE. It is important to note that EULAR and ACR intended these criteria to be used as a classification tool rather than a diagnostic criterion. However, it remains a helpful tool for clinicians in suspecting a diagnosis of SLE. Details of the EULAR/ACR 2019 criteria are summarized in Table 4 and Table 5 [1].

**Table 4:** European League Against Rheumatism/American College of Rheumatology (EULAR/ACR) clinical domains and criteria for systemic lupus erythematosus Antinuclear antibodies (ANA) must be present at a titer of ≥1:80 on HPe-2 cells, or an equivalent positive test is required. The additive criteria follow a positive ANA; the presence of at least one clinical criterion and a total score of 10 is required for classification as SLE. Within each domain, only the highest weighted criterion is counted toward the score.

Clinical Domains and Criteria	Criteria	Weight
Constitutional	Fever	2
Hematologic	Leukopenia	3
	Thrombocytopenia	4
	Autoimmune hemolysis	4
Neuropsychiatric	Delirium	2
	Psychosis	3
	Seizure	5
Mucocutaneous	Non-scarring alopecia	2
	Oral ulcers	2
	Subacute cutaneous or discoid lupus	4
	Acute cutaneous lupus	6
Serosal	Pleural or pericardial effusion	5
	Acute pericarditis	6
Musculoskeletal	Joint involvement	6
Renal	Proteinuria >0.5g/24h	4
	Renal biopsy class II or V lupus nephritis	8
	Renal biopsy class III or IV lupus nephritis	10

**Table 5:** European League Against Rheumatism/American College of Rheumatology (EULAR/ACR) immunological domains and criteria for systemic lupus erythematosus Antinuclear antibodies (ANA) must be present at a titer of ≥1:80 on HPe-2 cells, or an equivalent positive test is required. The additive criteria follow a positive ANA; the presence of at least one clinical criterion and a total score of 10 is required for classification as systemic lupus erythematosus (SLE). Within each domain, only the highest weighted criterion is counted toward the score. Anti-β2GPI:  Anti-β2 glycoprotein I

Immunological Domains and Criteria	Criteria	Weight
Antiphospholipid antibodies	Anti-cardiolipin antibodies OR Anti- β2GPI antibodies OR Lupus anticoagulant	2
Complement proteins	Low C3 OR low C4	3
	Low C3 AND low C4	4
SLE specific antibodies	Anti-dsDNA antibody OR Anti-smith antibody	6

Biomarkers

Biomarkers play a vital role in diagnosing SLE, assessing disease activity, classifying complications, and assessing disease response to therapeutic interventions. However, the clinical heterogeneity and the complex pathogenesis of SLE make it challenging for one biomarker to reflect the disease's state accurately. Additionally, no single biomarker has shown the ideal sensitivity and specificity for SLE; hence a combination of biomarkers reflecting different aspects of disease manifestations may be more effective in assessing SLE [59].

Antinuclear Antibody

ANA is usually seen in SLE like other immunological diseases and can be used for screening, diagnosis, and prognosis. As a biomarker of SLE, ANA has a high sensitivity ranging from 95% to 97% but low specificity of 20%. High levels of ANA can be seen in several other disorders, as well as a significant proportion of the healthy population; hence a positive ANA does not confirm the diagnosis of SLE, but a negative ANA makes it less likely [60]. Immunofluorescence assay (IF) is the gold standard test for ANA; although enzyme-linked immunosorbent assays (ELISAs) and multiplex assays are widely available, they lack sensitivity and are, therefore, less preferred over IF [61].

C3 and C4

Complement activation is a critical component of SLE pathogenesis, and measuring levels of C3 and C4 have been a standard component of laboratory evaluation to help assess disease activity in patients with SLE. Patients with low levels of C3 or C4, combined with a positive ANA test, have 94.3% specificity for an SLE diagnosis. In comparison, patients with simultaneously low C3 and C4 levels and a positive ANA test have 97.6% specificity for an SLE diagnosis [62]. However, owing to the low specificity of C3 and C4 when used in isolation, their reliability as biomarkers for SLE can be limited [63]. Recent studies suggest that elevated levels of plasma complement split products and cell-bound activation products are more useful diagnostic markers and closely correlate with SLE disease activity [64].

Anti-dsDNA

As one of the most distinct ANA types, anti-dsDNA antibodies have a high specificity (96%) for SLE and are the highest weighted criterion in the immunologic domain of the 2019 EULAR/ACR classification. The presence of anti-dsDNA antibodies has been correlated with renal involvement, as demonstrated by their deposition in glomeruli, basement membrane, and mesangium in SLE patients with active nephritis, thus proving to be a valuable marker to predict the development of LN. Anti-dsDNA antibodies are closely correlated to disease activity, and their levels can fluctuate over time. Therefore, levels can be undetectable during treatment and increase during a flare, especially in active nephritis. Due to this transient appearance of anti-dsDNA antibodies, their diagnostic sensitivity is low (52% to 70%) [1,65].

Anti-Smith Antibody

The presence of anti-Smith antibodies (anti-Sm), like anti-dsDNA antibodies, is the highest weighted criterion in the immunological domain in EULAR/ACR 2019 classification for SLE. Anti-Sm antibodies are highly specific for SLE, with a specificity of 99% [66]. Anti-Sm antibodies correlate with SLE disease activity and show a relatively static expression in peripheral blood, unlike anti-dsDNA antibodies, which show fluctuations in disease activity. Anti-Sm antibodies respond more slowly to changes in disease activity in SLE, implicating its use as a biomarker to assess disease activity in new-onset SLE. Moreover, anti-Sm antibodies are associated with lupus nephritis and have been identified as a predictor of silent LN and high disease activity, represented by lymphopenia and hypocomplementemia [67,68]. 

Anti-Ro (SSA) and Anti-La (SSB) Antibodies

Anti-Ro antibodies are seen in up to 50% of cases of SLE, and anti-La antibodies in up to 20%. These antibodies are highly associated with Sjögren syndrome with 90% specificity and can be used to assess secondary Sjögren syndrome in patients with SLE. However, they are also associated with subacute cutaneous lupus, photosensitivity, and neonatal lupus [69,70].

Urinary Biomarkers

Twenty-four-hour urine protein and protein/creatinine ratio are conventional urinary biomarkers for LN. Various urine protein biomarkers, including chemokines (monocyte chemoattractant protein-1, interferon-γ-inducible protein 10, and interleukin-8), cytokines (urinary tumor necrosis factor-like weak inducer of apoptosis, interleukin 17, interleukin-6, transforming growth factor-beta, adiponectin, and osteoprotegerin), adhesion molecules, and growth factors have been evaluated as potential SLE biomarkers, particularly for LN. However, none have approval for commercial use in clinical practice. Only a few have been independently validated [71].

Despite the performance of the EULAR/ACR criteria, some patients with SLE can still be misdiagnosed. This gap can be attributed to the lack of reliable biomarkers with an ideal sensitivity and specificity for SLE, the high level of physician skill and experience required to reach an accurate diagnosis, and the fact that few patients with SLE show clinical symptoms in the early stages of the disease.

Management

Systemic lupus erythematosus is a disease of heterogenic manifestation involving multiple organs; therefore, the disease severity and organ involvement vary from patient to patient, thus posing a significant challenge in disease management and requiring an interdisciplinary approach. The treatment aims to prevent the flare-ups of the disease, promote remission and maintenance, and prevent relapse at a minimum cost of side effects of the drugs used [72].

The choice of drugs used to treat the disease depends on the disease's activity. Since the EULAR guidelines for the management of SLE were published in 2008, there have been excellent advancements in managing the disease. Various scoring systems are used to assess the disease activity, among which the widely accepted are the Systemic Lupus Erythematosus Disease Activity Index-2000 (SLEDAI-2K), the Systemic Lupus Activity Questionnaire (SLAQ), Physician Global Assessment (PGA), the British Isles Lupus Assessment Group (BILAG 2004 index) and Lupus Foundation of America Rapid Evaluation of Activity in Lupus (LFA-REAL) [73]. In addition, since the damage done due to inflammation in SLE to various organs is irreversible, various indices are used to assess the damage, i.e., Systemic Lupus International Collaborating Clinics (SLICC), American College of Rheumatology Damage Index, and the Brief Index of Lupus Damage [74].

These scoring systems play a significant role in determining the choice of drugs and their effectiveness in disease management as a SLEDAI score of zero indicates complete remission or absence of any active inflammation, a SLEDAI score of 1-5 indicates mild disease activity, and SLEDAI score of 6-10 indicates moderate disease activity, an increase of SLEDAI score of 3 or more than 3 indicates flare-up of disease and decrease of a score of 3 or more indicates a response to the treatment and improvement in the disease activity [74].

Goal of Treatment

The goal of the treatment is to get optimum control of symptoms and remission of the disease, improve long-term outcomes for the patient, prevent end organ damage, and better quality of life for the patient [75]. The definition of an acceptable remission state in SLE includes a SLEDAI score of 3 or less than three antimalarial, PGA of 1 or less than 1 with less than or equal to 7.5 mg of prednisone. Therefore, the first line of drugs for the SLE maintenance phase is antimalarial, the most common of which is hydroxychloroquine. In acute phases, intravenous prednisolone is used, which might be later tapered off gradually and replaced by an immunosuppressant or antimalarial, depending on the disease severity [76].

Hydroxychloroquine is the first line of drug used in most patients. Response to hydroxychloroquine is promising; however, long-term therapy's associated risk of retinal toxicity requires patient monitoring. The risk increases once the duration of treatment exceeds five years or the dose exceeds 100 mg daily. Dose adjustment is required in preexisting retinal, macular, or chronic kidney disease. Considerable evidence from studies suggests that the risk of retinopathy can be minimized if the daily dose is less than 5 mg per kg body weight [77].

Glucocorticoids are usually used in the flare-up of the disease, and the aim is to achieve remission while tapering the dose and replacing it with hydroxychloroquine or immunosuppressant. The minimum tolerated dose is equal to or less than 7.5 mg daily to have minimal adverse effects. High doses of intravenous methylprednisolone 250-1000 mg/day for three days can be used in acute end-organ damage. The flare-ups of the disease and subsequent use of glucocorticoids to control can be minimized by the early introduction of immunosuppressants, considering the poor control of the disease by antimalarial alone [78,79].

Immunosuppressants are the choice of drugs when patients present with frequent relapse despite hydroxychloroquine or fail to respond to hydroxychloroquine, and the symptoms do not improve. Immunosuppressive has a significant role in disease flare-ups when a rapid tapering of glucocorticoids is desired. However, the choice of immunosuppressive depends on the symptoms, age, and family planning. Certain drugs are not safe during or before pregnancy and even need to be stopped before planning for pregnancy, thus requiring robust contraceptive protection [80].

Methotrexate and azathioprine should be considered when a patient fails to respond to the maximum safe dose of glucocorticoids and hydroxychloroquine. Others included are mycophenolate mofetil and cyclophosphamide, usually used in organ-threatening and rescue therapy in resistant cases [81].

Monoclonal Antibodies 

Belimumab is a human monoclonal antibody that prevents the binding of BlymS (B lymphocyte stimulator) to receptors on B cells, thus inhibiting the growth and proliferation of B cells into plasma cells, therefore, reducing the auto reactivity effect. The other drug used in this category is rituximab, a genetically structured monoclonal antibody directed against CD20 B cells. It inhibits B cell proliferation and thus prevents its differentiation into plasma cells [82]. Monoclonal antibodies are also considered an important treatment option when there is extra-renal disease manifestation with insufficient response to the first-line treatment, including a combination of hydroxychloroquine and prednisone with or without immunosuppressive therapy and failure of remission due to tapering off glucocorticoid to a dose of equal or less than 7.5 mg per day. Suitable candidates for monoclonal antibodies are those with very high disease activity, such as SLEDAI score of more than 10, prednisone dose of greater than 7.5 mg/day, the serological finding of low C3/C4 and high anti-dsDNA titers, and extra-renal manifestations.

Monoclonal antibodies are generally considered after failure to respond to maximum tolerable doses of immunosuppressive therapy [83]. Recently an anti-BDCA2 antibody named litifilimab has shown a significant role in the reduction of swollen and tender joints in SLE in phase 2 trials for 24 weeks. However, more extensive studies (such as randomized controlled phase 3 trials) are needed for a better understanding of the safety and efficacy of this intervention [84].

Since non-steroidal anti-inflammatory drugs (NSAIDs) are used as anti-inflammatory, antipyretic, and analgesic agents, they inhibit COX enzymes, thus reducing the synthesis of prostaglandins involved in the inflammatory process. Thus, NSAIDs are used in patients with serositis and musculoskeletal symptoms. In addition to NSAIDs, low-dose aspirin is also used to prevent thrombotic events in patients who carry anti-phospholipid antibodies [85,86].

Additional Treatment Options

Non-pharmacological therapies in treating SLE include intravenous immunoglobulin (IVIG) and plasmapheresis. The IVIG is believed to inhibit type 1 IFN-mediated differentiation, suppress the B cell activation, and neutralize the antibodies produced by the B cells. IVIGs are used in severe cases unresponsive to immunosuppressive drugs in patients with active SLE and concomitant infection. IVIG has effectively treated cutaneous and neuropsychiatric systemic lupus erythematosus [87,88].

Plasmapheresis is a purification technique in which the extracorporeal separation of blood components results in filtered plasma products used to separate autoantibodies and immune complexes, thus reducing the damage to the target organs. Plasmapheresis is used in patients with life-threatening manifestations of SLE, such as neuropsychiatric flare-ups. Complications and side effects of plasmapheresis are well understood since the procedure has been used over decades for several other diseases; however, the clinical benefit and cost-effectiveness of plasmapheresis is yet a question compared to using immunosuppressants and biological agents since the literature available is based on observation and case reports [89].

Initial and Maintenance Phase of Non-renal SLE

Initially, the target should be to control the active disease activity. The choice of drugs depends on disease intensity and the involvement of the organ. In case of mild cutaneous involvement, avoidance of sunlight, and use of sunscreen, topical glucocorticoid or topical tacrolimus can be used. Moderate involvement of joints and pleural membrane can be treated with a combination of NSAIDs and glucocorticoids during the initiation phase and later tapered off, gradually replacing it with immunosuppressive therapy. Since immunosuppressive therapy takes a long time to start its action, therefore, it should be introduced as soon as possible during the tapering phase of steroids. In severe cases, intravenous glucocorticoids can be used as initial therapy and later replaced with immunosuppressive or biological agents [72].

Refractory Non-renal SLE

For non-renal SLE, which is resistant to conventional therapy, the choice of drug is belimumab which is very effective in the case of resistant SLE but takes a longer duration for its action. The drug requires continuous monitoring of the disease activity through widely accepted scoring systems, episodes of flare-ups, and the requirement of steroids to control the active disease. Belimumab is of great significance in treating resistant cases; however, it still requires approval for its use in various countries [82,83].

Management of Lupus Nephritis

SLE affects the kidneys in almost 50% of patients, known as lupus nephritis, which results in chronic kidney disease and end-stage renal disease, ultimately resulting in death. The main problem with lupus nephritis is that by the time the problem gets clinically evident, it has already severally involved the kidneys; thus, the patients are treated with an anti-inflammatory immediately followed by a potent immunosuppressive agent. The induction phase of the treatment is usually three to six months, followed by a maintenance phase that lasts for three years. Detailed management of severe proliferative lupus nephritis is given in Table 6 [90].

**Table 6:** Management of severe proliferative lupus nephritis

Severe proliferative lupus nephritis	Induction Phase	Maintenance Phase
YES	Intravenous methylprednisolone 0.25-1g/day for 1-3 days followed by oral prednisolone 1mg/kg body weight to a maximum of 80mg/day, tapers over weeks, PLUS Intravenous cyclophosphamide 0.5-1g/m2 monthly for 6 months	Prednisone 5-10mg/day PLUS Mycophenolate mofetil 1-2g/day or Azathioprine 1-2.5mg/kg/day or Cyclosporine 2.5mg/kg/day if MMF/AZA not tolerated
NO	Oral prednisolone 1mg/kg/day to a maximum of 30mg/day tapper over weeks PLUS Low dose intravenous cyclophosphamide 500mg every 2 weeks for 3 months OR Oral cyclophosphamide 1-1.5 mg/kg/day to a maximum of 150 mg/day for 2-4 months OR Oral mycophenolate mofetil 2-3g/day for 6 months	The maintenance phase is the same as above

## Conclusions

The management and long-term complications of lupus have markedly improved in the last two decades, but still, there are a lot of unanswered questions regarding the disease. Current research is on its way, and further randomized control trials are needed in order to develop targeted therapies. Management of chronic diseases, especially lupus, requires excellent compliance and commitment. Poor drug compliance, lack of monitoring, and reassessing the disease activity usually lead to poor outcomes, frequent relapses, and resistance cases. Patients should be counseled regarding the disease pathology, signs and symptoms, the need for regular monitoring, medication compliance, and preventive measures such as lifestyle changes, dietary modification, and regular exercise to control weight and lipid levels and prevent cardiovascular complications.
